# Identification of the *PLA2G6* c.1579G>A Missense Mutation in Papillon Dog Neuroaxonal Dystrophy Using Whole Exome Sequencing Analysis

**DOI:** 10.1371/journal.pone.0169002

**Published:** 2017-01-20

**Authors:** Masaya Tsuboi, Manabu Watanabe, Kazumi Nibe, Natsuko Yoshimi, Akihisa Kato, Masahiro Sakaguchi, Osamu Yamato, Miyuu Tanaka, Mitsuru Kuwamura, Kazuya Kushida, Takashi Ishikura, Tomoyuki Harada, James Kenn Chambers, Sumio Sugano, Kazuyuki Uchida, Hiroyuki Nakayama

**Affiliations:** 1 Laboratory of Veterinary Pathology, Graduate School of Agricultural and Life Sciences, The University of Tokyo, Tokyo, Japan; 2 Laboratory of Functional Genomics, Graduate School of Frontier Sciences, The University of Tokyo, Tokyo, Japan; 3 Japan Animal Referral Medical Center, Kanagawa, Japan; 4 D&C Veterinary Clinic, Ibaraki, Japan; 5 Laboratory of Veterinary Microbiology I, School of Veterinary Medicine, Azabu University, Kanagawa, Japan; 6 Laboratory of Clinical Pathology, Joint Faculty of Veterinary Medicine, Kagoshima University, Kagoshima, Japan; 7 Laboratory of Veterinary Pathology, Graduate School of Life and Environmental Sciences, Osaka Prefecture University, Osaka, Japan; 8 Thermo Fisher Scientific, Life Technologies Japan Ltd., Tokyo, Japan; German Cancer Research Center (DKFZ), GERMANY

## Abstract

Whole exome sequencing (WES) has become a common tool for identifying genetic causes of human inherited disorders, and it has also recently been applied to canine genome research. We conducted WES analysis of neuroaxonal dystrophy (NAD), a neurodegenerative disease that sporadically occurs worldwide in Papillon dogs. The disease is considered an autosomal recessive monogenic disease, which is histopathologically characterized by severe axonal swelling, known as “spheroids,” throughout the nervous system. By sequencing all eleven DNA samples from one NAD-affected Papillon dog and her parents, two unrelated NAD-affected Papillon dogs, and six unaffected control Papillon dogs, we identified 10 candidate mutations. Among them, three candidates were determined to be “deleterious” by *in silico* pathogenesis evaluation. By subsequent massive screening by TaqMan genotyping analysis, only the *PLA2G6* c.1579G>A mutation had an association with the presence or absence of the disease, suggesting that it may be a causal mutation of canine NAD. As a human homologue of this gene is a causative gene for infantile neuroaxonal dystrophy, this canine phenotype may serve as a good animal model for human disease. The results of this study also indicate that WES analysis is a powerful tool for exploring canine hereditary diseases, especially in rare monogenic hereditary diseases.

## Introduction

Whole exome sequencing (WES), a technique for sequencing all the exons of protein-coding genes, has become a powerful and common tool for identifying causative genes of inherited disorders. Since the first isolation of a causative mutation in 2010 [1], more than 150 mutations of human inherited disorders have been identified by WES analysis [2]. When compared with whole genome sequencing, WES requires less monetary cost and results in a higher quality of sequence coverage, as it targets only 1 to 2 per cent of the total genome. Recently, the application of WES analysis has also been expanded to several domestic species, as the genomic databases of non-human animals have become more accurate. The genome sequence database for domestic dogs (*Canis familiaris*) has been updated and improved several times since the first publication of a high-quality draft genome sequence in 2005 [3]. The WES enrichment kit based on the latest version of the canine reference genome, CanFam3.1 [4], was designed in 2014 [4], making it much easier to identify genetic mutations in inherited diseases [5] [6].

Neuroaxonal dystrophy (NAD) is a group of rare, heterogeneous inherited neurodegenerative disorders that has been described in various mammalian species, including humans [7–8], dogs [9–21], sheep [22–24], cattle [25], horses [26–28], cats [29–30], rabbits [31], rats [32], and mice [33–37]. Although they all share a characteristic pathological feature, *i*.*e*., “spheroids” in the central and, rarely, peripheral nervous system, there is some variation in the onset of clinical symptoms and lesion distribution between and also within species [20]. Human infantile neuroaxonal dystrophy (INAD; OMIM 256600) or neurodegeneration with brain iron accumulation (NBIA) 2A, is one of the most common types of human NAD [7], while other types of NBIAs, such as pantothenate kinase-associated neurodegeneration (PKAN or NBIA1; OMIM 234200), mitochondrial membrane protein-associated neurodegeneration (MPAN or NBIA4; OMIM 614298), and β-propeller protein-associated neurodegeneration (BPAN or NBIA5; OMIM 300894) also represent histological hallmarks of NAD. After an initial gen@ome-wide linkage study in 12 families of INAD patients revealing a spectrum of mutations in the *PLA2G6* gene [38], a number of missense or frameshift mutations in the *PLA2G6* gene have been identified, accounting for approximately 85% of INAD patients [39].

In dogs, NAD has previously been reported in several breeds, such as Rottweilers [9–11], Collie sheepdogs [12], Papillons [13–16], Giant Schnauzer-Beagle crossbreeds [18], Jack Russell terriers [19], Spanish Water dogs [20], and a Dachshund-cross dog [21]. Among these, genetic studies have been extensively performed for a few breeds, and only two forms of canine NAD have been characterized at the molecular level: a mitofusin 2 (MFN2) missense mutation with foetal onset NAD in laboratory dogs [18] (Giant Schnauzer-Beagle crossbreed) and a TECPR2 missense mutation in Spanish Water dogs [20]. However, the causal mutations of the other forms of canine NAD remain unclear.

In the present study, using WES analysis and TaqMan genotyping assays, we identified the *PLA2G6* c.1579G>A missense mutation in Papillon dog NAD.

## Results

### Clinical manifestations of neuroaxonal dystrophy in papillon dogs

Three affected Papillon puppies were referred to veterinary hospitals, and general and neurological examinations were conducted.

**Fig 1:**
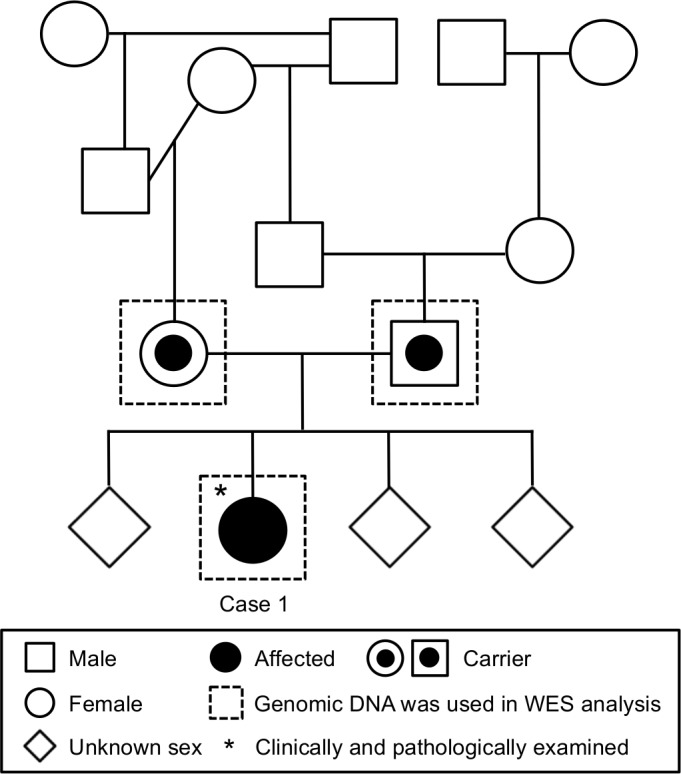
Pedigree analysis of Case 1. Some paternal and maternal individuals were repetitively used for breeding, resulting in an increase in genetic homogeneity.

Case 1: The dog had shown a wobbling gait since 4 months of age and suffered astasia starting one month after the initial symptoms. At 6 months of age, neurological symptoms, including blindness, loss of menace response, and strabismus, gradually developed (S1 Video). MRI examination revealed mild cerebellar atrophy, while other regions of the nervous system appeared normal. At 7 months of age, the dog had difficulty eating, leading to euthanasia at the owner’s request. The results of pedigree analysis are shown in Fig 1. As identical individuals have been frequently used for breeding, increased genetic homogeneity was predicted in the offspring.

Case 2: The dog could not walk until 2 months of age and appeared to be blind, leading the owner to bring the dog to a veterinary hospital at 3 months of age. Intension tremor, limb extension, and astasia were observed upon initial examination. There were no abnormalities in the results of a blood test, X-ray examination, or deep pain test. Steroid treatment was not effective. At 4 months of age, the dog was referred to the Veterinary Medical Center of Osaka Prefecture University for a more detailed investigation. MRI examination revealed mild cerebellar atrophy but no significant lesions in the other regions of the nervous system. As the dog exhibited a poor prognosis, he was euthanized at the owner’s request.

Case 3: The dog had already demonstrated head tremors at the time of purchase and collapsed at 3 months of age. The dog then had hind limb paresis, which further extended to the forelimbs. The dog was referred to Kagoshima University Veterinary Teaching Hospital. However, there were no conclusive MRI findings. Due to its breed, age of onset, and the symptoms, the case was suspected to be NAD. However, further information was not obtained, as the owner did not request any further investigation.

### Pathological examination of neuroaxonal dystrophy in papillon dogs

**Fig 2:**
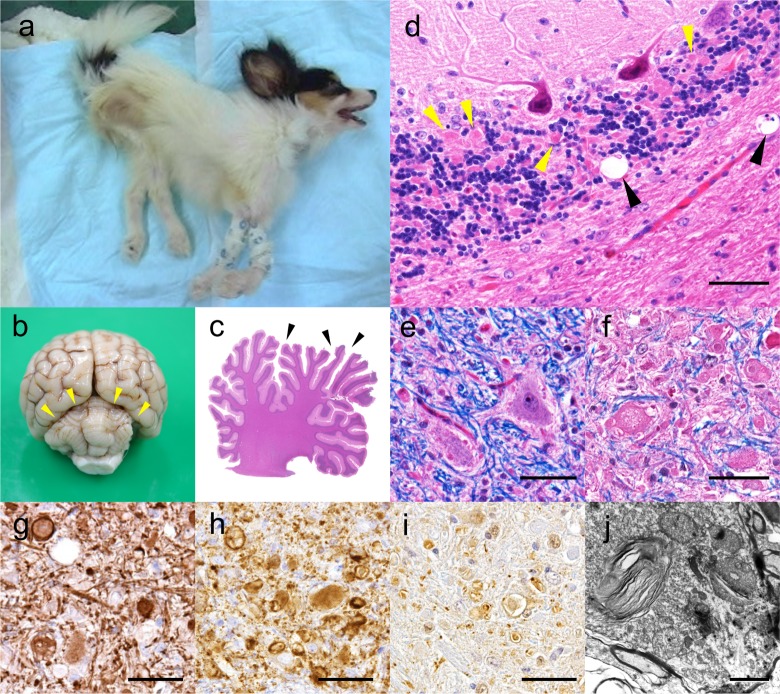
Clinical and pathological findings of NAD in Papillon dogs. a) Case 1. The dog showed persistent intention tremor and was severely emaciated due to eating difficulty. The appendicular muscles were atrophied. b) Photographic example of the formalin-fixed brain of Case 1. The cerebellum shows mild atrophy (yellow arrowheads). c) Sagittal section of the cerebellum of Case 2. The cerebellar cortex is mildly atrophied and the sulci are dilated (arrowheads). HE stain. d) Numerous small to medium-sized spheroids (yellow arrowheads) are observed in the cerebellar granular layer. Vacuolization is also observed at the border of the granular layer and white matter (black arrowheads). HE-stain. Bar = 50 μm. e) The olivary nucleus of Case 1. Small to medium-sized spheroids are scattered around the neurons. LFB-HE stain. Bar = 50 μm. f). The cuneate nucleus of Case 1. Medium-sized to large spheroids are frequently observed. The spheroids are eosinophilic and granular, and some contain clefts and vacuoles in the core. LFB-HE stain. Bar = 50 μm. g) to i) The cuneate nucleus of Case 1. Bar = 50 μm. NF (g) and synaptophysin (h) immunohistochemistry showed strong reactivity in the axonal spheroids. i) Ubiquitin immunohistochemistry showed numerous granularly positive spheroids. The surrounding small spheroids are also strongly positive. j) Transmission electron microscopy of a spheroid in the dorsal horn of the spinal cord. The spheroid contains dense membranous material and swollen mitochondria. Bar = 2 μm.

Pathological examination was performed on the brains of two NAD-affected Papillon dogs (Cases 1 and 2). Case 1 showed severe emaciation, and the appendicular muscles were atrophied (Fig 2A). The cerebellar vermis showed mild atrophy (Fig 2B), while the cerebrum and spinal cord appeared intact. Mild dilation of the third and fourth ventricles was also observed (data not shown).

Histopathologically, a number of axonal spheroids were observed throughout the central nervous system, including the thalamus, hippocampus, mesencephalon, cerebellar cortex, cerebellar nuclei, medulla oblongata, and dorsal horn of the spinal cord in both cases (Fig 2D–2F). The axonal spheroids were eosinophilic, round to oval in shape, and varied in size from 5 to 50 μm in diameter. Some large spheroids contained clefts, granules, or vacuoles in the core. Immunohistochemical analysis revealed that the spheroids were strongly positive for neurofilaments (Fig 2G) and synaptophysin (Fig 2H). Faint to moderate-sized granules positive for ubiquitin were also observed in the spheroids (Fig 2I). Small to large vacuoles were frequently observed around large spheroids (Fig 2D and 2F). The cerebellar cortex appeared to be atrophied, and the cerebellar sulcus was dilated (Fig 2C). The number of Purkinje cells in the molecular layer of the cerebellum was moderately decreased. Mild gliosis was observed in the neuropil of the molecular layer. A few torpedoes were detected in the cerebellar white matter. In contrast, the cerebral cortex appeared intact. Iron deposits were not detected in the brain. Transmission electron microscopic observation of the dorsal root of the spinal cord revealed dense membranous materials and numerous swollen mitochondria in the large spheroid (Fig 2J). A few spheroids were also observed in the optic nerve of Case 1 (data not shown). Spheroids were not observed in the other sensory, somatic, or autonomic nerves. All other organs appeared pathologically intact in Cases 1 and 2.

### Whole exome sequencing and bioinformatics analysis

WES was performed for the three NAD-affected Papillon dogs, parents of Case 1, and six unaffected control Papillon dogs (S2 Table). Nucleotide sequence data are available in the DDBJ Sequenced Read Archive (http://trace.ddbj.nig.ac.jp/dra/index_e.html) under the accession numbers DRX061214- DRX061235. A total of 90 to 135 million DNA fragments were mapped to the reference genome (CanFam3.1), with average genome coverage of 3.09 to 4.63. In an *in silico* study, 60,000 to 80,000 of a total of 122,652 SNPs and 6,000 to 8,000 of a total of 17,851 indels were detected. Among them, exon-coding mutations that matched the criteria were selected; *i*.*e*., the homozygous mutations in NAD-affected cases, heterozygous mutations in NAD parents, and no homozygous mutations in controls, with ≥10x coverage depth (the selection procedure is summarized in Table 1). There were ten missense mutations that matched our criteria, while no indel mutations matched the criteria. The ten selected candidate mutations are shown in Table 2.

**Table 1:** Number of SNPs and indel mutations identified through the WES analysis and candidate selection.

SNP filtering step	Number of SNPs	Indel filtering step	Number of indels
Total SNPs	173,849	Total indels	17,851
Coverage ≥10x SNPs	122,652	Coverage ≥ 10x indels	14,030
Gene coding SNPs	97,734	Mutated in all NAD and NAD-parents	2,124
Exon coding SNPs	46,639	NAD homozygous indels	135
NAD homozygous and NAD-parent heterozygous SNPs	59	NAD-parent heterozygous indels	5
Control wild-type or heterozygous SNPs	18	Control wild-type or heterozygous indels	2
Missense mutations	10	Exon coding indels	0

**Table 2:** Overview of all variants identified by exome sequencing and *in silico* pathogenicity analysis.

Gene annotation	Chromosome	Position	Mutation		Computational predictions		
			mRNA level	Protein level	PolyPhen-2	PROVEAN	SIFT
*SLC35E3*	10	10,905,842	c.1363A>C	p.R145S	0.001	-0.404	0.400
Novel Gene (ENSCAFG00000029271)	10	23,266,184	c.130G>A	p.K44E	0.617 *	-1.000	0.320
*L3MBTL2*	10	24,002,628	c.2711A>G	p.R675Q	0.000	-0.263	1.000
*XPNPEP3*	10	24,347,707	c.725G>A	p.H242R	0.003	-1.239	0.290
*PDGFB_CANFA*	10	25,817,180	c.693C>A	p.E231D	0.048	-0.740	0.030 **
*PLA2G6*	10	26,544,757	c.1516G>A	p.I506V	0.000	-0.437	0.190
*PLA2G6*	10	26,544,820	c.1579G>A	p.T527A	0.201	-2.403	0.010 **
*CCDC66*	20	33,717,816	c.2024T>C	p.T675M	0.011	-1.007	0.720
*PGBD1*	35	25,421,942	c.38G>A	p.D13G	0.000	0.227	0.880
*ZSCAN31*	35	25,456,698	c.412G>A	p.T138A	0.141	0.158	0.450

* possibly damaging.

** deleterious.

All the extracted mutations were analysed using the *in silico* pathogenicity prediction tools PolyPhen-2, SIFT, and PROVEAN. The respective scores are summarized in Table 2. Using the PolyPhen-2 prediction, only the novel gene (ENSCAFG00000029271) c.130G>A mutation was predicted as “possibly damaging”, while the other mutations were “benign”. Using the SIFT prediction, the *PDGFB_CANFA* c.693C>A and *PLA2G6* c.1579G>A mutations were predicted as “deleterious”, while the others were “tolerant”. The PROVEAN prediction demonstrated that all mutations were predicted as “benign”; however, the score of the *PLA2G6* c.1579G>A mutation was the closest to the threshold score of -2.5.

### TaqMan SNP genotyping assay

A large-scale TaqMan SNP genotyping assay for mutations with a high pathogenicity score (ENSCAFG00000029271 c.130G>A, *PDGFB_CANFA* c.693C>A and *PLA2G6* c.1579G>A) were conducted for 61 dogs, including 3 NAD cases, 2 relatives, and 57 unaffected control Papillon dogs (S1A Fig). Despite its low pathogenicity score, a genotyping assay of the *PLA2G6* c.1516G>A mutation was also performed, as its homologue is a candidate gene for human INAD.

In the ENSCAFG00000029271 c.130G>A, *PDGFB_CANFA* c.693C>A and *PLA2G6* c.1516G>A mutations, allelic discrimination plots of the unaffected dogs were distributed in various areas, including homozygous A/A (upper-left area), heterozygous mutant G/A (upper-right area), and wild type G/G (lower-right area). By contrast, in the *PLA2G6* c.1579G>A mutation, the plots of all the control dogs were distributed in the wild type. As only the *PLA2G6* c.1579G>A mutation had an association with the presence or absence of the disease, we assumed that this mutation might be a causal mutation of canine NAD.

Additional *PLA2G6* c.1579G>A genotyping was conducted for an NAD affected dog (mix of Papillon and Chihuahua, previously reported by Nibe *et al*. [10]), 82 unaffected Papillons, 49 unaffected Chihuahuas, an unaffected Papillon-Chihuahua mix, and 118 unaffected dogs of other breeds (details are listed in S3 Table)). All of the unaffected dogs tested possessed a G/G wild-type allele, while an additional NAD-affected dog was homozygous A/A (Table 3).

**Table 3:** Results of TaqMan genotyping assay for the PLA2G6 c.1579G>A mutation in Papillon, Chihuahua and other breeds.

Breed		Cases tested	Wild-type [G/G]	Hetero [G/A]	Homo [A/A]
	NAD-affected Papillon	3	0	0	3
	NAD-affected Papillon x Chihuahua mix	1	0	0	1
	NAD carrier Papillon	2	0	2	0
	Unaffected Papillon	82	82	0	0
	Unaffected Chihuahua	49	49	0	0
	Unaffected Papillon x Chihuahua mix	1	1	0	0
	Unaffected other breeds	118	118	0	0
Total		256	250	2	4

### Direct sanger sequencing

To validate the identified *PLA2G6* c.1579G>A mutation, we re-sequenced the genomic DNA encompassing the mutated alleles using direct Sanger sequencing and confirmed that the affected cases were homozygous A/A, the carriers were heterozygous G/A, and the controls had the wild-type G/G allele (all the controls tested in this study were homozygous for the wild-type alleles) (S1B Fig).

### Comparative bioinformatic sequence analysis of iPLA_2_β

**Fig 3:**
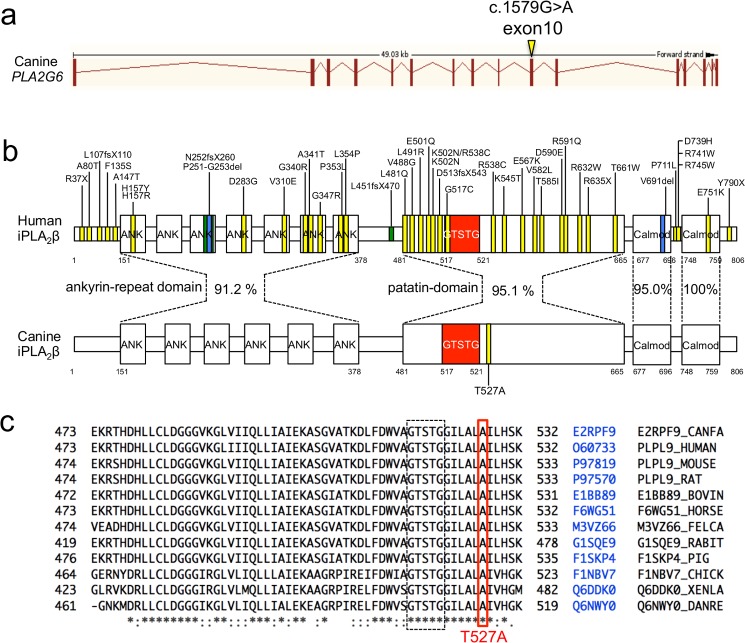
Structures of the *PLA2G6* transcript and iPLA_2_β protein. a) Structure of the canine *PLA2G6* gene. The *PLA2G6* gene contains 16 exons with a total length of 49.03 kb. The identified mutation in this study, c.1579G>A, was located at exon 10. b) Protein structure of human and canine iPLA_2_β and the homology of the ankyrin-repeat domain, patatin domain, and two calmodulin-binding domains. ANK; ankyrin domain Calmod; calmodulin domain and GTSTG; lipase motif. Yellow and blue bands in human iPLA_2_β indicate known substitutions and deletion mutations in human INAD, respectively. The yellow band in the canine iPLA_2_β indicates the T526A mutation identified in this study. c) ClustalW multiple protein alignment of the region of iPLA_2_β containing the mutation. The target alanine is shown in the red box. Sequences for dogs (E2RPF9_CANFA), humans (PLPL9_HUMAN), mice (PLPL9_MOUSE), rats (PLPL9_RAT), cows (E1B889_BOVIN), horses (F6WG51_HORSE), cats (F6WG51_FELCA), rabbits (G1SQE9_RABIT), pigs (F1SKP4_PIG), chickens (F1NBV7_CHICK), African clawed frogs (Q6DDK0_XENLA) and zebrafish (Q6NWY0_DANRE) are shown. The target alanine lies within a region of 13 highly conserved amino acids that contains the GTSTG lipase domain.

*PLA2G6* mRNA construction and iPLA_2_β protein sequences were obtained from the Ensembl Genome Browser (http://www.ensembl.org/index.html) and UNIPROT (http://www.uniprot.org/), respectively, and differences in the protein sequences among mammalian species were compared. The *PLA2G6* mRNA (ENSCAFT00000002213.3) consisted of 16 exons. The mutation was located at exon 10, resulting in a threonine substitution at the 527^th^ amino acid (p. Ala527Thr) of canine iPLA_2_β (E2RPF9_CANFA) (Fig 3A). Both human (PLPL9_HUMAN) and canine iPLA_2_β consisted of 806 amino acids and had N-terminal ankyrin-repeat domains, a patatin domain, and C-terminal calmodulin-binding domains (Fig 3B). There were seven repeating ankyrin domains in humans, while only six repeats were predicted in dogs. The homology between full-length human and canine iPLA_2_β was 90.8%. The homology of the ankyrin-repeat domain, patatin domain and two calmodulin domains between the species was 91.2%, 95.1%, 95.0%, and 100%, respectively.

The identified mutation is located near the serine lipase consensus sequence (GTSTG) of the patatin domain. Multiple alignment revealed high conservation of the amino acid sequences near the GTSTG serine lipase sequence in vertebrate animals, including dogs, humans, mice (PLPL9_MOUSE), rats (PLPL9_RAT), cows (E1B889_BOVIN), horses (F6WG51_HORSE), cats (F6WG51_FELCA), rabbits (G1SQE9_RABIT), pigs (F1SKP4_PIG), chickens (F1NBV7_CHICK), African clawed frogs (Q6DDK0_XENLA) and zebrafish (Q6NWY0_DANRE), and the 527th alanine was unchanged in these species (Fig 3C).

### iPLA_2_β immunohistochemistry

**Fig 4:**
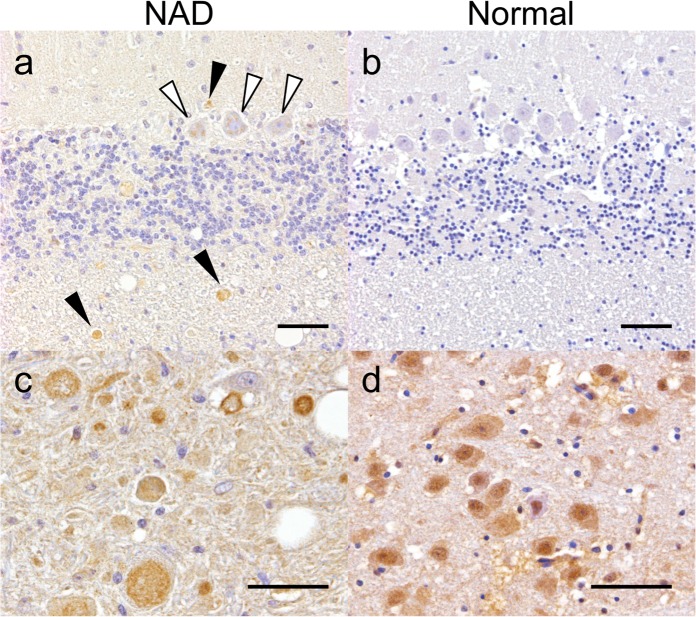
iPLA_2_β immunohistochemistry of Case 1 (a, c) and a normal (b, d) canine brain. (a) Spheroids in the cerebellum showed faint granular expression (black arrowheads). Cytoplasmic granular expression was also observed in the Purkinje cells (white arrowheads). (b) The expression was very weak in the cerebellum of the control dog. In the brainstem (c, d), intense cytoplasmic and nuclear expression was detected in the neurons of both the NAD and control dogs. Axonal spheroids in the neuropil of the brainstem of Case 1 showed an intense granular expression (arrowheads). Bar = 50 μm.

Immunohistochemistry for iPLA_2_β was performed in NAD-affected and control Papillon dogs (Fig 4). In the control dogs, strong cytoplasmic and nuclear expression of the protein was detected in the neurons of the brainstem (Fig 4D), but the expression intensity was lower in the neurons of the cerebral and cerebellar cortexes (Fig 4B). NAD-affected cases showed similar expression patterns of the iPLA_2_β protein in the brainstem. In addition, granular to patchy positive iPLA_2_β expression was observed in the small to large axonal spheroids of the brainstem (Fig 4A) and cerebellum (Fig 4C). Moreover, the cytoplasm of some Purkinje cells was faintly positive for iPLA_2_β (Fig 4A, arrowheads).

## Discussion

Clinical and/or pathological manifestations of the present three NAD cases were consistent with those in previous reports. In Papillon dogs, the disease was first reported in 1995 in England [13], and since then, it has expanded to Japan [14–16] and Canada [17]. The affected dogs initially developed intention tremor and hypermetria at a very young age, and the symptoms gradually progressed to cerebellar ataxia, tetraplegia, blindness, and deafness. Histopathologically, multiple spheroid formation is observed throughout the central nervous system, including the cerebrum, hippocampus, thalamus, mesencephalon, cerebellum, pons, medulla oblongata, and dorsal horn of the spinal cord, while the peripheral nerves are generally unaffected. As all the reported cases consider analogous time points of disease onset (13–16 weeks of age) and death (usually 7 or 8 months of age), it is assumed to be an inherited disease, with monogenic autosomal recessive inheritance [14].

We succeeded in collecting parent-offspring trio samples from an affected Papillon family and were able to perform genetic analysis using a next generation sequencer. As we sought to obtain “disease-specific” variants and rule out “Papillon-specific variants” from the candidates, we used Papillon genomes as control samples. By analysing a total of eleven Papillon dogs, we were able to narrow down the candidates to just 10 missense variants. Among these, 3 variants were predicted to be “deleterious” by *in silico* analysis, and an additional mass screening using a TaqMan genotyping assay revealed that the c.1579G>A missense mutation had an association with the presence or absence of the disease, suggesting its possibility as a causal mutation of NAD in Papillon dogs.

*PLA2G6* mutations have been previously identified in patients with INAD and adult-onset dystonia-Parkinsonism (PARK14; OMIM 612953). Most common mutations in INAD patients are single-base substitutions that result in amino acid alterations, but short (2370delTG [40], 2070_2072delTGT [38, 41]) and long (6.6kb-del from introns 4 to 6 [42]) nucleotide deletions have also been reported. To date, more than 40 positions in the *PLA2G6* gene have been identified as causes of INAD. The mutated positions vary; some mutations are located in the patatin-like phospholipase domain, others in the ankyrin-repeat domain, and there are additional mutations in other regions. The *PLA2G6* mutation has also been identified in a mouse model of INAD (*PLA2G6-inad*), which was generated by N-ethyl-N-nitrosourea (ENU) mutagenesis [37]. This mouse model bears a point mutation in the ankyrin domain (p.Gly373Arg) and shows spheroid formation throughout the nervous system from an early age.

A novel c.1579G>A missense mutation of canine *PLA2G6* in the present study results in p.Ala527Thr substitution of canine iPLA_2_β. iPLA_2_β is a phospholipase that hydrolyses the *sn*-2 ester bond of membrane phospholipids, resulting in the release of arachidonic acid (AA; 20:4(n-6)) and docosahexaenoic acid (DHA; 22:6(n-3)). There is a high level of homology between the human and canine iPLA_2_β proteins, especially in the patatin domain and the two calmodulin domains. While the mutated amino acid in canine iPLA_2_β is located near the serine lipase consensus sequence “GTSTG” of the patatin domain and is highly conserved among a number of animals, we suspect that its lipase activity may be impaired, as in some human *PLA2G6* mutations [43].

In contrast to NAD in Papillon dogs, human INAD appears to have a wide histopathological spectrum. Previous studies of human INAD revealed that 87% (34 of 39) of *PLA2G6* mutation-positive patients presented with spheroid formation in the peripheral nerves (including the skin, conjunctiva, sural nerve, muscle, areola, and rectal mucosa) in addition to the central nervous system, and only 48% (14 of 29) of patients presented iron accumulation [40]. Some INAD patients present Lewy bodies and neurofibrillary tangles in the brain, suggesting a shared pathogenesis with Parkinson’s and Alzheimer’s diseases [44]. This histopathological diversity may be caused by different mutation positions of *PLA2G6*, although the consensus between the mutations and their phenotypes has not yet been determined. As there is no evidence of peripheral nerve involvement or brain iron accumulation in Papillon NAD, it may serve as an animal model for the non-PNS-involved and non-iron-deposited variants of human INAD. Differences in biological predispositions among humans and non-human animals should be taken into consideration along with the other pathological features of neurodegenerative diseases (*i*.*e*., senile plaques or neurofibrillary tangles) [45–46].

The precise mechanism of how dysfunction of *PLA2G6* leads to axonal degeneration still remains unclear. Interestingly, there was strong iPLA_2_β expression in the brains of NAD-affected dogs, especially in the axonal spheroids. Although the reason for this iPLA_2_β distribution pattern also remains unknown, the mutated iPLA_2_β seems to participate in the pathogenesis of spheroid formation. Double immunofluorescence analysis for detecting ubiquitin and iPLA_2_β was conducted; however, no marked co-localization was observed in the axonal spheroids (data not shown). This result indicates that the causal relationship between the gene mutation and axonal spheroid formation is complicated. Previous reports have demonstrated iPLA_2_β expression in various regions of the brain [47–48]. Immunoreactivity to iPLA_2_β in normal monkeys was observed throughout the brain, including in the midbrain, cerebellum, thalamus, hypothalamus, and caudate nucleus [49], where axonal spheroids are also usually observed in NAD dogs. A recent study of *PLA2G6*-deficient mice indicated that the specific degeneration of the mitochondrial inner membrane and the synaptic membrane occurs before spheroid formation [50]. This study also revealed an increased presence of degenerated mitochondria at the distal axon during the early presymptomatic stage and that mitochondria are disrupted during the later stage of motor dysfunction. This may cause the release of reactive oxygen species and cytochrome c outside the ruptured mitochondria and possibly result in neuronal apoptosis.

## Conclusion

By combining the results of WES and TaqMan genotyping analyses, we identified a novel candidate causal mutation of canine NAD, *PLA2G6* c.1579G>A, in Papillon dogs. As a homologue of this gene is a causative gene for human INAD, this canine counterpart may serve as a good animal model for human disease and can be utilized to explore treatments for both human and canine NAD Furthermore, WES analysis enabled us to narrow down the mutations to just 10 candidates using only eleven DNA samples (one trio set of parent-offspring dogs, two unrelated NAD-affected Papillon dogs, and six unaffected control Papillon dogs), indicating that this method of analysis can be a powerful tool in studying canine hereditary diseases, especially in monogenic hereditary conditions.

## Materials and Methods

### Sample collection and processing

DNA samples for WES examination were collected from the blood and/or cerebellar tissue of 3 NAD Papillon dogs, the 2 parental dogs of each affected case, and 6 Papillon dogs without any clinical signs of NAD. Genomic DNA was purified using the QIAGEN DNA Blood & Tissue Mini Kit (QIAGEN, Hilden, Germany) according to the manufacturer’s protocol. Genomic DNA yield and quality were determined using a Nanodrop ND1000 spectrophotometer (Thermo Fisher Scientific, Waltham, MA) and a Qubit dsDNA BR Assay kit (Life Technologies, Invitrogen division, Darmstadt, Germany). Additional DNA samples for the TaqMan genotyping assay were obtained from the Azabu University canine DNA genomic bank and Kagoshima University Veterinary Teaching Hospital.

### Histopathology and immunohistochemistry

Two of the three NAD cases were autopsied following euthanasia. Tissue samples were collected from the entire body, including the brain and spinal cord. Samples were fixed in 10% neutral-buffered formalin, processed routinely, embedded in paraffin wax, and then sectioned into 4-μm sections. Sections were stained with haematoxylin and eosin (HE) or with luxol-fast blue followed by HE (LFB-HE).

Immunohistochemistry was performed in selected samples using the Envision polymer reagent (Dako-Japan, Kyoto, Japan). After deparaffinization, the sections were incubated with 3% hydrogen peroxide (H_2_O_2_)-methanol at room temperature for 5 minutes, 12% skim milk-tris buffered saline (TBS) solution at 37°C for 30 minutes to avoid non-specific reaction, and finally, primary antibody at 4°C overnight. Primary antibodies were mouse monoclonal anti-neurofilament (NF) antibody (clone 2F11, pre-diluted; Dako-Japan), mouse monoclonal anti-synaptophysin antibody (clone SY38, x50; Dako-Japan), rabbit polyclonal anti-ubiquitin antibody (x100; Dako-Japan), and rabbit polyclonal anti-iPLA_2_β antibody (x200; Bios Inc., Woburn, MA). For iPLA_2_β and synaptophysin immunohistochemistry, antigen retrieval was performed before H_2_O_2_-methanol incubation by heating in a pH 6.0 citrate buffer at 121°C for 10 minutes.

After primary antibody incubation, sections were rinsed with TBS and then incubated with Envision horseradish peroxidase (HRP)-labelled anti-mouse or rabbit IgG polymer (Dako) at 37°C for 60 minutes. Visualization of the reacted products was conducted with 3.3’-diaminobenzidine (DAB, Dojindo, Kumamoto, Japan) and 0.03% H_2_O_2_ in TBS, and then counterstaining was conducted with Mayer’s haematoxylin. Negative controls were performed by omitting primary antibodies.

### Electron microscopy

Formalin-fixed brain tissue from the brainstem of Case 1 was cut into 1-mm cubes, fixed in 2% glutaraldehyde in 0.1 M phosphate buffer (pH 7.4), and then post-fixed in 1% osmium tetroxide in 0.1 M cacodylate buffer (pH 7.2) at 4°C for 2 hours. Tissues were then dehydrated through a graded series of ethanol, replaced with QY-1 solution (Nisshin EM, Tokyo, Japan), and embedded in an epoxy resin (Quetol 651, Nisshin EM, Tokyo, Japan). Ultrathin sections were stained with uranyl acetate and lead citrate and examined with a Hitachi H-7500 transmission electron microscope (Hitachi High-Technologies, Tokyo, Japan).

### DNA preparation for WES

Whole exome libraries of NAD-affected and control samples were constructed using the SureSelect Canine All Exon Target Enrichment System (Agilent Technologies, Santa Clara, CA) according to the manufacturer’s protocol. DNA samples (3 μg) were sheared into 150-bp fragments by sonication using a Covaris S2 (Covaris, Woburn, MA). The ends of DNA fragments were repaired using T4 DNA polymerase, Klenow DNA polymerase, and T4 polynucleotide kinase; then, single A bases were added at the 3’ ends by Exo(-) Klenow DNA polymerase. SureSelect LTI5500 P1 and 1A adaptors were ligated to the A-tailed DNA fragments using T4 DNA ligase. Between each procedure, the DNA samples were purified using Agencourt’s AMPure XP beads (Beckman Coulter, CA). After adapter ligation, the DNA samples were amplified by 5 cycles of PCR using Herculase II Fusion DNA polymerase. After amplification, the DNA concentration and their base lengths were determined using the Agilent 2100 Bioanalyzer (Agilent Technologies) and then concentrated to 221 ng/μl by centrifugation.

### Target enrichment and next generation sequencing

Target enrichment was performed using the SureSelect Canine All Exon Target Enrichment System (Agilent Technologies). DNA samples were hybridized with a biotin-labelled RNA capture library (bait), which was designed for canine whole exome sequences. Approximately 750 ng of the DNA samples was added to Agilent blocking reagents, denatured at 95°C, and incubated at 65°C. Hybridization buffer was added to the bait, and the total aliquot was added to the heated DNA library. The DNA-library-bait mixture was hybridized at 65°C for 24 hours. After hybridization, the DNA-RNA hybrids were purified using the Dynabeads MyOne Streptavidin T1 system (Life Technologies). After purification, the samples were amplified by 8 cycles of PCR using Herculase II Fusion DNA polymerase and purified using Agencourt’s AMPure XP beads. The concentration of the purified samples (exome library) was determined using both the Agilent 2100 Bioanalyzer and the SOLiD Library TaqMan Quantitation Kit (Life Technologies).

Next generation sequencing was performed using the SOLiD 5500xl System (Life Technologies) according to the manufacturer’s protocol. After diluting the exome libraries to 500 pM, emulsion PCR amplification was conducted using the SOLiD EZ Bead System (Life Technologies). The libraries were emulsified with equivalently concentrated P1 beads using the SOLiD EZ Bead Emulsifier, packed into the EZ Bead Amplifier Pouch, and then amplified with the SOLiD EZ Bead Amplifier. After amplification, the template beads were enriched using the SOLiD EZ Bead Enricher. The 3’ ends of the bead-bound DNA fragments were modified, and the beads were deposited into the SOLiD FlowChip. The FlowChip was then sent to the SOLiD 5500xl sequencer, and paired-end sequencing was performed.

### Bioinformatic analysis

Sequence analysis was conducted using SOLiD LifeScope software (Applied Biosystems). After quality checking, the sequence fragments were mapped onto the canine reference genome CanFam3.1 (http://www.ensembl.org/Canis_familiaris/Info/Index). SNP and short Indel mutations were extracted. Mutations were then filtered by coverage of ≥ x10, and mutations that caused amino acid alterations were extracted. As the disease was expected to expand in an autosomal recessive manner, we predicted that the cause of the disease would be a homozygous mutation in the affected cases and a heterozygous (or hemizygous) mutation in the parent cases. After mutations matching, such situations were filtered, and the online *in silico* prediction algorithms PolyPhen-2 (http://genetics.bwh.harvard.edu/pph2/), PROVEAN (http://provean.jcvi.org/index.php) and SIFT (http://sift.jcvi.org/) were used to estimate the pathogenicity of protein changes due to missense mutations.

### Comparative sequence analysis of iPLA_2_β

Protein sequences and other information on iPLA_2_β were obtained from UNIPROT (http://www.uniprot.org/). Sequence homology between human and canine iPLA_2_β was analysed using the Clustal Omega aligning programme. A multiple sequence alignment of iPLA_2_β was constructed using the CLUSTALW tool (http://www.genome.jp/tools/clustalw/).

### TaqMan SNP genotyping assay

Using the Custom TaqMan^®^ SNP Genotyping Assay Kit (Life Technologies), large-scale allele screening against the candidate mutations was performed to validate the mutant allele frequency in Papillon dogs. Probes and primers were designed using the Custom TaqMan^®^ Assay Design Tool (https://www.lifetechnologies.com/order/custom-genomic-products/tools/genotyping/). For each allele, FAM^TM^ and VIC^®^ were used as reporter dyes. The nucleic acid base sequences of the probes and the primers are summarized in S1 Table.

The Applied Biosystems 7900HT Fast Real-Time PCR System (Applied Biosystems) was used for PCR reactions. Samples (5 μl) containing 2 ng of genomic DNA, 2.5 μl of TaqMan Universal PCR Master Mix (2x), and 0.125 μl of Custom TaqMan SNP Genotyping Assay mix (x40) were put in a 384-well reaction plate, and thermocycling was performed under the following conditions: initial denaturation at 95°C for 10 minutes was followed by 40 cycles at 95°C for 15 seconds and 60°C for 1 minute. No template control (NTC) reactions were conducted with the same procedure, with the exception of excluding the genomic DNA. Thereafter, PCR plates were read on an SDS 7700 instrument (Applied Biosystems) using the end-point analysis mode of the SDS software package (ABI). The fluorescence intensity for each cycle product was plotted, and the amplification curve was drawn using the Rn (ratio of the reporter dye fluorescence signal intensity to the fluorescence intensity of the passive reference dye signal) of each cycle. Genotypes of homozygous, heterozygous and wild-type alleles were virtually determined using the dye-component fluorescent emission data illustrated in the X-Y scatter-plot of the SDS software.

### Direct sanger sequencing

Direct Sanger sequencing was performed to validate the *PLA2G6* c.1579G>A (Ensembl number: ENSCAFT00000002213) mutation. To include the DNA sequences 150 bp up- and down-stream of the identified *PLA2G6* mutation, forward (5’-TCCAGCTTCTCATCGCCATC-3’) and reverse (5’-CACCCCACAAAGGGCTTTCA-3’) primers were designed using Primer-BLAST online software (http://www.ncbi.nlm.nih.gov/tools/primer-blast/).

The PCR reaction was conducted in a volume of 25 μl containing 10 ng of sample genomic DNA, 8 μl of 2.5 mM dNTP mixture (TaKaRa, Otsu, Japan), 10 pmol of the forward and reverse primers, 0.2 μl of Phusion High Fidelity (HF) DNA Polymerase (Thermo Scientific), and 5 μl of Phusion 5x HF buffer (Thermo Scientific). Thermocycling was carried out as follows: initial denaturation at 98°C for 30 seconds, was followed by 35 cycles at 98°C for 30 seconds, 60°C for 30 seconds, and 72°C for 2 minutes, and finally at 72°C for the final extension before cooling at 4°C. The DNA amplicons were purified using the QIAGEN DNA purification kit (QIAGEN), loaded onto a 1% agarose gel, and then electrophoresed for 25 minutes. A 256-bp DNA band was excised from the gel and purified with the SV Gel and PCR Clean-Up System (Promega, Madison, WI, USA). Purified DNA samples were then directly sequenced using an ABI 3730xl DNA sequencer (Applied Biosystems, Foster City, CA, USA). Sequence data were analysed using an ABI 3130xl genetic analyser (Applied Biosystems).

### Ethics statement

As all the sampling procedures in this study were performed during the medical examination or after the necropsy, no ethics committee approval was required. However, all animal procedures were conducted according to the national guidelines for animal welfare in Japan (Act on the Welfare and Management of Animals).

All the dogs evaluated in this study were privately owned pets and were examined at the veterinary hospitals with the consent of their owners, including informed consent from owners approving use in research studies. Blood sampling of the dogs was performed in the veterinary clinics during the routine diagnosis of NAD. All necropsied dogs used in the study were euthanized at the owner’s request due to the severity of clinical signs, and their necropsies were performed with the owner’s permission. Euthanasia was performed by the intravenous injection of fentanyl and propofol, immediately followed by the rapid intravenous injection of potassium chloride. This procedure was performed by licensed veterinary clinicians of the respective veterinary hospitals.

## Supporting Information

S1 Fig TaqMan genotyping analysis of the four selected candidate mutations. a) Allelic discrimination plots of the four candidate genes. The plots are expressed as the endpoint Rn values of VIC and FAM for each allele at the X- and Y-axes. In the assay of the novel gene, *PDGFB_CANFA* and *PLA2G6* c.1516G>A mutations, plots of control Papillon dogs are distributed in all areas of homozygous mutated (upper left), heterozygous mutated (upper right), and wild-type (lower right) genes, indicating that some unaffected Papillon dogs have the homozygous mutated allele. However, the *PLA2G6* c.1579G>A mutation in all the normal Papillon dogs’ plots are distributed in the wild-type area (lower right). b) Sanger sequence of a portion of *PLA2G6* showing the G>A mutation at c.1579 (highlighted in red letters), resulting in an alanine to threonine substitution.
(TIF)

S1 Table Nucleic acid sequences of the probes and primers used in the TaqMan genotyping assay. (XLSX)

S2 Table Primary results of WES analysis. (XLSX)

S3 Table Numbers of dogs of other breeds used for TaqMan genotyping assays. (XLSX)

S1 Video Case 1 showing progressive intention tremor. (MP4)
