# Supplementary figures and images for: Identification of the PLA2G6 c.1579G>A Missense Mutation in Papillon Dog Neuroaxonal Dystrophy Using Whole Exome Sequencing Analysis
Source: PLoS One. 2017 Jan 20;12(1):e0169002. doi: 10.1371/journal.pone.0169002 (PMC5249094; doi:10.1371/journal.pone.0169002)

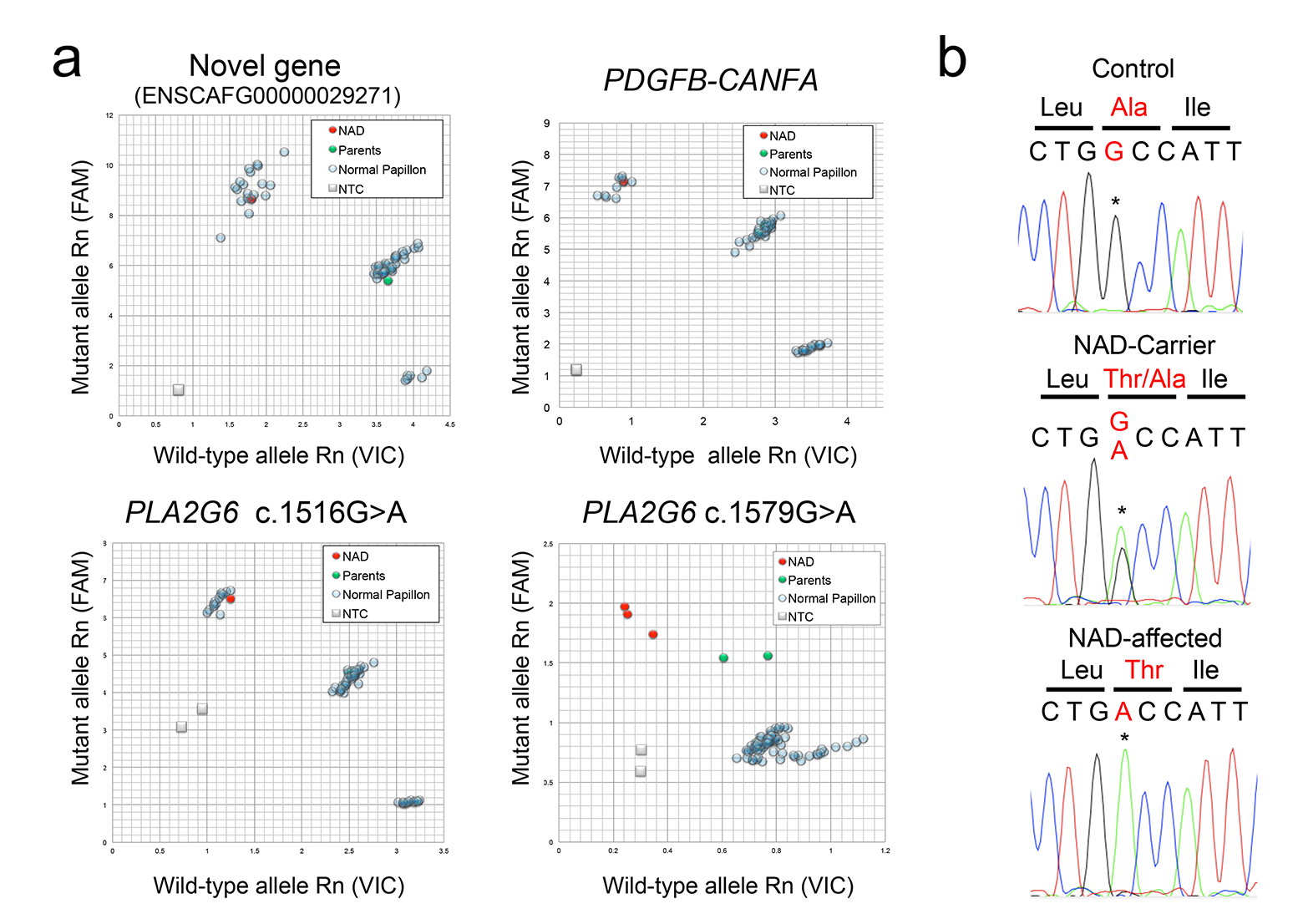

Supplement: S1 Fig — a) Allelic discrimination plots of the four candidate genes. The plots are expressed as the endpoint Rn values of VIC and FAM for each allele at the X- and Y-axes. In the assay of the novel gene, PDGFB_CANFA and PLA2G6 c.1516G>A mutations, plots of control Papillon dogs are distributed in all areas of homozygous mutated (upper left), heterozygous mutated (upper right), and wild-type (lower right) genes, indicating that some unaffected Papillon dogs have the homozygous mutated allele. However, the PLA2G6 c.1579G>A mutation in all the normal Papillon dogs’ plots are distributed in the wild-type area (lower right). b) Sanger sequence of a portion of PLA2G6 showing the G>A mutation at c.1579 (highlighted in red letters), resulting in an alanine to threonine substitution. (TIF) [file pone.0169002.s001.tif]
